# New Appliances and Things Medical

**Published:** 1909-02-06

**Authors:** 


### NEW APPLIANCES AND THINGS MEDICAL.

[We shall be glad to receive at our Office, 28 & 29 Southampton Street, Strand, London, W.C., from the manufacturers, specimens of all new preparations and appliances.]

### VALISAN.

(E. Schering and Co., Berlin.)

Valisan is a compound of valerian, camphor (Borneol), and bromine. It thus possesses the anti-spasmodic action of valerian and camphor, and at the same time the sedative actions of the bromides. From clinical observations it is evident that valisan is a useful sedative in many functional nervous disorders. It contains approximately 25 per cent. of bromine, 48 per cent. of borneol, and 26.5 per cent. of iso-valerianic acid. It compares favourably with regard to its taste with other similar drugs, such, for instance, as bornyval. The drug is put up in pearls, each containing 0.25 grammes. The dose is from one to three several times a day.

### NOVASPIRIN.

(F. Bayer and Co., Ebsterfeld, 19 St. Dunstan's Hill, London, E.C.)

Novaspirin is a preparation of which the active principle is salicylic acid. It does not possess the depressing effects of the ordinary salicylates, and its diaphoretic action is also less than other preparations of this nature, such, for instance, as aspirin or ordinary acetyl-salicylic acid. Chemically novaspirin is methylene-cytril-salicylic acid. It contains approximately 62 per cent. of salicylic acid; it is not decomposed in the stomach, and therefore is free from the occasional unpleasant gastric effects of the salicylates. In the intestine nascent salicylic acid is rapidly split off from it, and exerts its usual therapeutic effect, without being accompanied by any unpleasant by-effects. After this drug is taken by the mouth the urine gives a reaction, according to our experiments, for salicylic acid in about forty minutes. The average dose of the drug is 15 grains several times a day, and it is best given in tablets or cachets. We think the preparation is to be recommended.

### BROMURAL.

(Knoll and Co., London, E.C.)

This preparation is chemically a brom-iso-valerianyl-urea. The urea derivative which is most generally used as a hypnotic at the present day is veronal. The substance before us is a urea derivative containing bromine and valerian. Bromural is made up into tablets containing 5 grains each, and is given in doses of from one to two tablets. It is a mild hypnotic, and apparently produces no unpleasant after-effects. It has no cardiac depressant action, and is fairly rapidly absorbed, inducing sleep in cases of insomnia not due to pain in from half an hour to one hour after its administration. We have used the remedy to a considerable extent, and have found it efficacious in the above doses in mild cases of insomnia.

### LACTIC ACID FERMENT.

With reference to lactic acid ferment for the production of sour milk, we are informed by Oppenheimer, Son and Co., Ltd., of 179 Queen Victoria Street, E.C., that they keep a constant crop of the true bacilli growth. They acknowledge their indebtedness to Mr. Petko Taptchileshtoff, of Sofia, Professor Metchnikoff, and three well-known London professors for their assistance in working out a satisfactory method of preparation. They point out that these cultures, so far as they are able to discover, cannot be reduced to a condition of powder without contamination with other germs, and that it is desirable that only the true culture of the Bulgarian bacillus should be used. The makers send out this growth in boxes, each containing twelve tubes, at 4s. 6d. per box. The contents of each tube are sufficient for a pint of milk, and it is desirable that it should not be held in stock, but should be purchased fresh as required.

